# The Antiviral Effect of High-Molecular Weight Poly-Gamma-Glutamate against Newcastle Disease Virus on Murine Macrophage Cells

**DOI:** 10.1155/2014/301386

**Published:** 2014-12-30

**Authors:** Melbourne Talactac, Jong-Soo Lee, Hojin Moon, Mohammed Y. E. Chowdhury, Chul Joong Kim

**Affiliations:** ^1^College of Veterinary Medicine and Biomedical Sciences, Cavite State University, 4122 Cavite, Philippines; ^2^College of Veterinary Medicine, Chungnam National University, Daejeon 305-764, Republic of Korea; ^3^Chittagong Veterinary and Animal Sciences University, Chittagong 4202, Bangladesh

## Abstract

This study demonstrates the capacity of HM-*γ*-PGA treatment to significantly protect murine macrophage cells (RAW 264.7 cells) against NDV infection. Such protection can be explained by the induction of antiviral state of HM-*γ*-PGA in RAW 264.7 cells via TLR4-mediated IRF-3, IRF-7, IFN-*β*, and IFN-related gene induction as shown in time-dependent changes in mRNA expression confirmed by polymerase chain reaction (PCR). Moreover, the present research also showed that HM-*γ*-PGA can induce proinflammatory cytokine secretion in RAW 264.7 as measured by enzyme-linked immunosorbent assay (ELISA). Therefore, our findings suggest that HM-*γ*-PGA can be a potential antiviral substance that can inhibit NDV infection through its stimulation of antiviral state on RAW 264.7 cells. These results have been consistent with the previous studies showing that HM-*γ*-PGA can protect RAW 264.7 cells and mice against influenza infection. However, it should be noted that although murine macrophage cells are susceptible to NDV, they are not the natural host cells of the virus; thus further in vivo and in vitro studies involving chicken and chicken immune cells are needed to fully assess the efficacy and applicability of HM-*γ*-PGA in the poultry industry.

## 1. Introduction

Newcastle disease virus (NDV) is a member of the Paramyxoviridae family under the genus* Avulavirus* [1, 2] and is currently designated as avian paramyxovirus virus serotype 1 (APMV-1) [3]. According to the Office International des Epizooties (OIE) in 2009, NDV strains can be classified into five pathotypes according to the clinical signs shown by the affected chickens, namely, viscerotropic velogenic (high mortality and hemorrhagic intestinal lesions), neurotropic velogenic (high mortality, respiratory, and nervous signs), mesogenic (low mortality, respiratory signs with occasional nervous signs), lentogenic (subclinical or mild infection), and asymptomatic enteric (subclinical enteric infection) ones [4].

Newcastle disease remains prevalent worldwide, though a number of live and inactivated NDV vaccines are available to control the disease [5, 6]. However, the currently available commercial vaccines have their limitations and one of them is the absence of genetic markers for serological differentiation between vaccinated and naturally infected birds. There are also reports suggesting that the types of NDV strains that have been identified circulating in poultry already showed major antigenic drift. Thus, there is a need for better NDV vaccines which can solve such problems, wherein viral vector vaccines prove to be a good alternative [7], as exemplified by the first licensed commercial recombinant vaccine, a recombinant Newcastle disease virus vaccine, using fowl pox virus as the vector to express immunogenic proteins from the NDV [8]. However, recombinant vaccines are not widely used by the poultry industry due to their inability for use in population-based mass application procedures and high cost [5].

In addition, currently available vaccines cannot provide adequate immunity in poultry even with the use of multiple vaccinations and live vaccines do not completely prevent infection or virus shedding [7–9]; thus natural substances with the ability to inhibit or prevent NDV infections might provide much needed additional protection against the disease. One of the natural substances which is widely studied for its various biological functions and applications is the high-molecular-weight poly-*γ*-glutamate (HM-*γ*-PGA) (>3000 kDa), a natural, edible, and biodegradable polymer derived from* Bacillus subtilis* subsp.* chungkookjang* [10–12]. Recently, the antiviral function of HM-*γ*-PGA against influenza virus through stimulation of Type I interferon (IFN) and Mx1 proteins both* in vitro* and* in vivo* was demonstrated [13]. Additionally, several studies have shown that HM-*γ*-PGA is functionally better than low molecular weight poly-*γ*-glutamate (LM-*γ*-PGA) (10–1,000 kDa) when it comes to antitumor activity and immune stimulation and when used as an adjuvant [14, 15].

In this study, the antiviral effect of HM-*γ*-PGA against NDV was evaluated on murine macrophage cell line (RAW 264.7). Based on the results, this study has shown that HM-*γ*-PGA protects murine macrophage cells from NDV infection through Type I interferon induction.

## 2. Methodology

### 2.1. Preparation of HM-*γ*-PGA

Endotoxin-free poly-*γ*-glutamate (*γ*-PGA) produced from* Bacillus subtilis* subsp.* chungkookjang* was prepared and provided by BioLeaders Corporation (Daejeon, Korea) in 0.85% sterile NaCl solution. Briefly, the culture broth of* B. subtilis* subsp.* chungkookjang* was collected and mixed with 3 times volume of ethanol. The precipitate was lyophilized and reconstituted in 10 mM Tris-HCl buffer (pH 7.5), treated with proteinase K, and dialyzed in distilled water. Next, the *γ*-PGA was purified by anion-exchange chromatography and dialyzed using Sep-Pak Plus Waters Accell Plus QMA cartridge (Millipore, USA) equilibrated with distilled water. Next, the cartridge column charged with *γ*-PGA was stepwise developed with NaCl solutions from 0.1 to 1.0 M. By estimating the concentration of glutamate in hydrolyzed *γ*-PGA using an amino acid analyzer, the content of *γ*-PGA was calculated by the following formula: content of *γ*-PGA (%) = (amount of glutamate/amount of sample) × (*A*/*B*) × 100.* A* = 129 (molecular mass of *γ*-glutamyl residue in *γ*-PGA);* B* = 147 (molecular mass of glutamate). The number and weight-average molecular masses (M*n* and M*w*, resp.) along with the polydispersity (M*w*/M*n*) of *γ*-PGA molecules were measured by gel permeation chromatography using a GMPWXL column (Viscotek, USA) and a LR125 Laser Refractometer (Viscotek, USA). Polyacrylamide standards (American Polymer Standard, USA) were used to construct a calibration curve and polydispersity of high-molecular weight *γ*-PGA was measured. The content of high-molecular mass *γ*-PGA was increased to >99%, and polydispersity was decreased after anion-exchange chromatography. To thoroughly get solubilized *γ*-PGA, the pH was adjusted to 7.0 by adding 5N sodium hydroxide (NaOH) solution to the acid form of PGA. Only purified HM-*γ*-PGA was used in this study [16].

### 2.2. Cell Culture and Virus

RAW 264.7 cells (ATCC TIB-71) were maintained in Dulbecco's Modified Eagles Minimum essential medium (DMEM) (Invitrogen, USA) containing 10% fetal bovine serum (FBS) (Gibco, USA) and 1% antibiotic/antimycotic (Gibco, USA) at 37°C with 5% CO_2_ until use.

The NDV-green fluorescence protein (NDV-GFP) was kindly provided by Dr. J. Jung of University of Southern California. The virus was amplified in 9- to 10-day-old embryonated eggs. Collected allantoic fluid was ultracentrifuged at 20,000 ×g for 2.5 hours at 8°C in 20% sucrose solution for viral concentration. Viral pellet was resuspended in PBS and 500 ul aliquots were kept at −70°C until use.

### 2.3. Antiviral Assay

To evaluate the antiviral effect of HM-*γ*-PGA against NDV infection, the inhibition of virus replication was examined. Briefly, RAW 264.7 cells were cultured with a suitable number (8 × 10^5^ cells/well) onto 12-well tissue culture (TC) plates (Nunc, Denmark) for 12 hours. Then, the medium was substituted with DMEM (for w/o treatment and medium treated cells) and DMEM with 1 mg/mL HM-*γ*-PGA. DMEM with 1000 units of recombinant mouse interferon-*β* (Sigma, USA) served as the positive control. After 12 hours of incubation, cells were infected with 1 multiplicity of infection (MOI) of NDV-GFP virus. The GFP expression was observed at 200x magnification 12 hours after infection (hpi) [13].

Cell viability was carried out via trypan blue exclusion test as described elsewhere. Briefly, RAW 264.7 cells were treated with 1 mg/mL of HM-*γ*-PGA, recombinant mouse interferon-*β*, or DMEM only (NC and medium treated cells) for 12 hours. After incubation, the cells were infected with NDV-GFP virus and cell viability was determined by trypan blue exclusion test at 30 hpi. Clarified cells from each group were mixed with 0.4% trypan blue stain (Invitrogen, USA) at 1 : 1 ratio. After staining, 10 *μ*L of the mixture was applied to a hemocytometer, wherein to get the percentage of viable cells, the total number of viable/live cells per mL of aliquot was divided by the total number of cells/mL of aliquot multiplied by 100. Cell counting was done thrice.

Lastly, following the instructions of the manufacturer, the GFP expression levels of medium only, 1 mg/mL HM-*γ*-PGA, and IFN-*β* treated cells 12 h before NDV-GFP infection were measured 24 hpi using Glomax multidetection system (Promega, USA). Briefly, the cells from each treatment group as described above were collected separately and centrifuged at 1200 rpm for 3 minutes. The resulting cell pellet was diluted in PBS and transferred to 96-well black plate for GFP detection. The test was done in triplicate [17].

### 2.4. NDV-GFP mRNA Expression on RAW 264.7 Cells

Total mRNA from RAW 264.7 cells was extracted and amplified in order to estimate the NDV-GFP mRNA expression [18]. Briefly, cells (8 × 10^5^/well) were cultured on 12-well TC plates (Nunc, Denmark) for 12 hours. Then, the medium was replaced with DMEM (for w/o treatment and medium treated cells) and DMEM with 1 mg/mL HM-*γ*-PGA. After 12 hours of incubation, cells were infected with 1 MOI of NDV-GFP virus. Cells were collected at 0, 6, 12, and 24 hours after infection. Total mRNA was extracted from RAW 264.7 cells using the RNeasy Mini Kit (Qiagen, USA). Reverse transcription of the total mRNA was carried out using M-MLV Reverse Transcriptase (Enzynomics, Korea), oligo (dT) 16-primers, and dNTP (0.5 *μ*M). M-MLV Reverse Transcriptase was incubated at 72°C for 5 minutes and 37°C for 60 minutes and terminally inactivated by heating at 72°C for 15 minutes. The polymerase chain reactions (PCR) using specific primers for Matrix gene of APMV-1 [19] and murine glyceraldehyde 3-phosphate dehydrogenase (GAPDH) housekeeping gene (internal control) [20] were carried out using emerald PCR master mix (Takara, USA) with 1 pM of each primer set (Table 1). The cDNA was amplified for 30 cycles with optimized annealing temperature for each primer set. Final extension was performed at 72°C for 5 minutes. Equal amounts of PCR products were run on 1.5% ethidium bromide impregnated agarose gels and visualized using GelDoc Imaging System (Bio-Rad, USA). On the other hand, relative band intensity (RBI) of the Matrix gene and GAPDH mRNA expression was determined using GelDoc Imaging System Band Quantification Software (Bio-Rad, USA).

### 2.5. Stimulation of an Antiviral State by HM-*γ*-PGA Treatment

To confirm the upregulation in mRNA expression level of antiviral related genes on RAW 264.7 cells, the method of Moon et al. [13] was used with some modifications. Briefly, the RAW 264.7 cells were seeded to each well of a 6-well TC plate (Nunc, Denmark) and cultured for 12 hours. Separately, the cells were treated with 1 mg/mL of HM-*γ*-PGA in DMEM with 1% FBS, 100 ng/mL LPS in DMEM as the positive control, and DMEM with 1% FBS only as the negative control. The treated cells were harvested at 0, 3, 6, 12, and 24 hours posttreatment. Afterward, preparation of total RNA and RT-PCR was performed using the same method above. The specific primers for PCR are listed in Table 2 [13, 20, 21].

### 2.6. Detection of Proinflammatory Cytokines Induced by HM-*γ*-PGA on RAW 264.7 Cells by ELISA

The proinflammatory cytokine inducing effect of HM-*γ*-PGA on RAW 264.7 cells was examined using commercial ELISA kits for murine interleukin-6 (IL-6), interleukin-12 (IL-12), tumor necrosis factor-alpha (TNF-*α*) (BD Bioscience, USA), and murine interferon-*β* (IFN-*β*) (PBL Interferon Source, USA) [13, 22, 23]. Briefly, a suitable number (2 × 10^6^/well) of RAW 264.7 cells were seeded to each well of a 6-well TC plate (Nunc, Denmark) and cultured. Twelve hours later, the culture medium was removed and the cell monolayer was washed once with PBS. The cells were then treated with 100 ng/mL of lipopolysaccharide (LPS) derived from* E. coli* 0111:B4 (Sigma-Aldrich, USA) or 1 mg/mL of HM-*γ*-PGA in DMEM with 1% FBS and incubated at 37°C with 5% CO_2_. Cells treated with DMEM with 1% FBS only served as negative control. Supernatant from each treatment group was harvested at 0, 6, 12, 24, and 36 hours posttreatment and clarified by centrifugation at 2500 ×g for 10 minutes at 4°C. Clarified supernatant was dispensed into the murine IFN-*β* ELISA plate for the measurement of secreted murine IFN-*β*, while 10-fold diluted supernatant was dispensed into the mouse TNF-*α*, IL-6, and IL-12 capture antibody coated ELISA plate. The test was performed in triplicate.

### 2.7. Statistical Analysis

Differences between groups were analyzed by Student's *t*-test. *P* values less than 0.05 were regarded as significant and those less than 0.01 were regarded as highly significant.

## 3. Results

### 3.1. Inhibition of Virus Replication by HM-*γ*-PGA on RAW 264.7 Cells

Previous studies revealed that HM-*γ*-PGA has beneficial functions to immune responses [12, 14, 16]. Recent studies also confirmed the role of *γ*-PGA in inducing cytokines involved in antiviral states in cells, leading to inhibition of virus replication [13].

**Figure 1:**
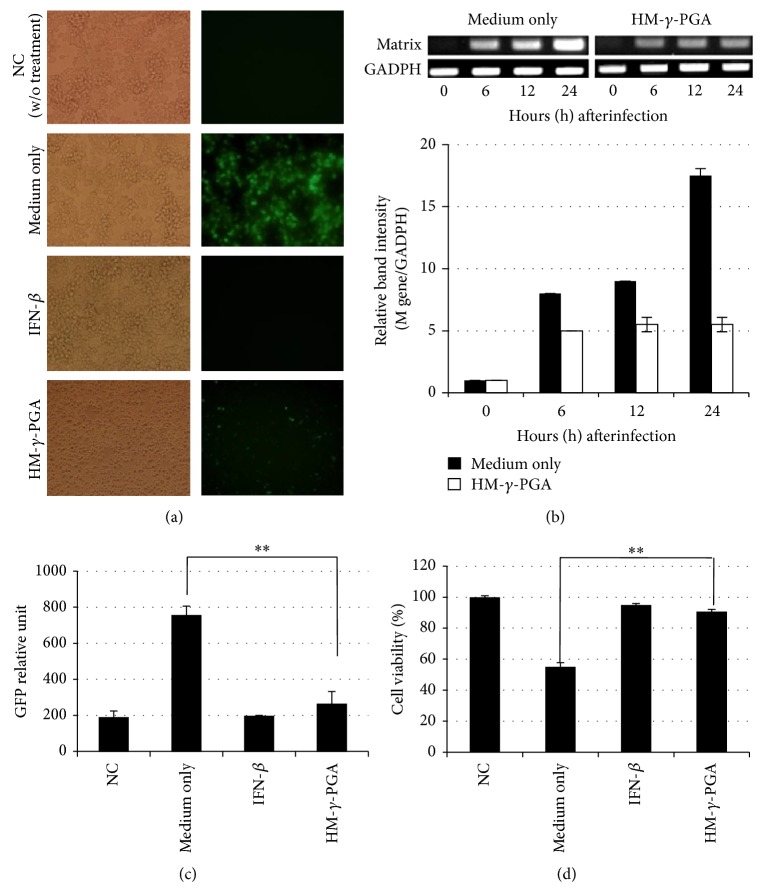
Antiviral function of HM-*γ*-PGA in murine macrophage cell line. (a) GFP expression images of medium only, 1 mg/mL HM-*γ*-PGA, and 1000 units/mL recombinant mouse IFN-*β* treated cells 12 h before NDV-GFP infection. Images taken 12 hpi (200x magnification). (b1) Viral mRNA expression level of Matrix gene of NDV-gfp over time in each treatment group was confirmed by specific RT-PCR primers which are shown in Table 1. Equal amounts of PCR products were run on 1.5% ethidium bromide impregnated agarose gels and visualized using GelDoc Imaging System. All samples were normalized using their respective GAPDH gene expression. (b2) Relative band intensity (RBI) of the Matrix gene mRNA expression of (b1). RBI was determined (Gene/GAPDH) using GelDoc Imaging System Band Quantification Software. Error bars indicate the range of values obtained from two independent experiments. (c) GFP expression level of media treated, 1 mg/mL HM-*γ*-PGA, and IFN-*β* treated cells 12 h before NDV-GFP (La Sota strain) infection. GFP expression was measured 24 hpi using Glomax multidetection system. (d) Cell viability was determined by trypan blue exclusion test at 30 hpi. The results are presented as a percentage of the control (cells without treatment). Error bars on Figures (c) and (d) indicate the range of values obtained from triplicate counting (^**^ *P* < 0.001, highly significant difference).

In this study, HM-*γ*-PGA treated RAW 264.7 cells showed markedly reduced virus replication while the medium treated cells demonstrated high level of GFP expression (Figures 1(a) and 1(c)). HM-*γ*-PGA treated cells showed a significant twofold reduction of GFP expression compared to medium treated cells.

Likewise, after virus infection, HM-*γ*-PGA treated cells showed less than 10% cell death while medium treated cells showed more than 50% cell death at 30 hpi (Figure 1(d)). Additionally, with the failure of the NDV-GFP virus to bud successfully from RAW 264.7 cells, we opted to measure the mRNA expression of the Matrix gene of the virus via RT-PCR to estimate virus replication (Figure 1(b)). As expected, the M gene expression of the HM-*γ*-PGA treated cells is relatively lower than the medium treated cells from 6 to 24 hours after infection. The medium treated cells also showed continuous expression of the M gene up to 24 hours after infection while HM-*γ*-PGA treated cells demonstrated nonincreasing pattern beginning after 12 hours.

### 3.2. Induction of Antiviral Genes and Proinflammatory Cytokines by HM-*γ*-PGA in RAW 264.7 Cells

To determine the mechanism by which HM-*γ*-PGA induces antiviral state in RAW 264.7 cells, the antiviral related gene expression and secreted proinflammatory cytokines from the murine macrophage cells were confirmed after HM-*γ*-PGA stimulation. RAW 264.7 cells were treated with 1 mg/mL of HM-*γ*-PGA and compared with 100 ng/mL of LPS treated cells.

**Figure 2:**
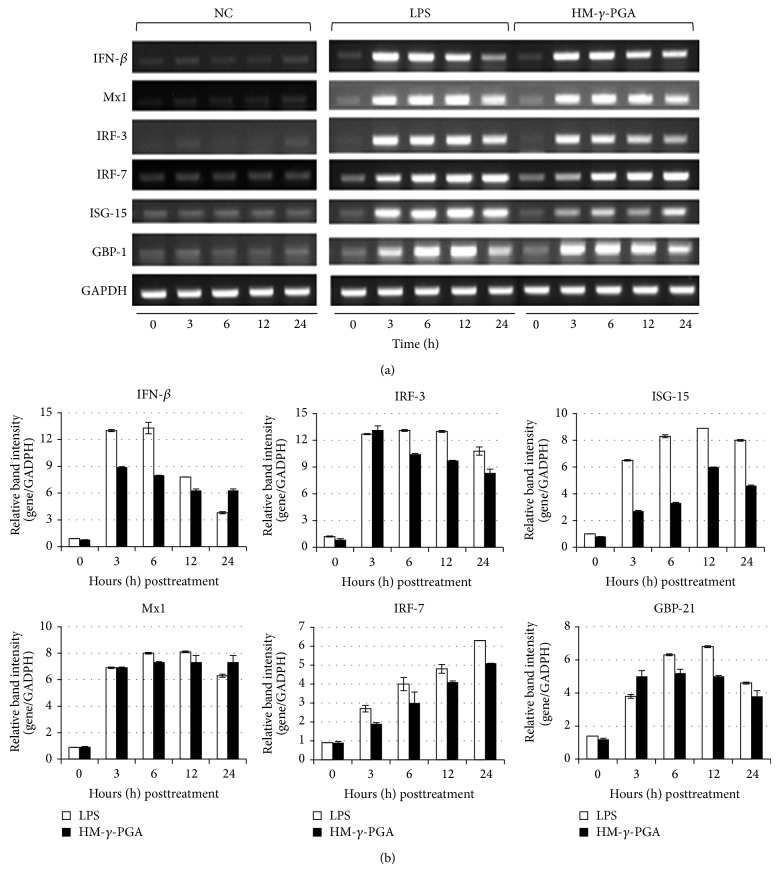
Induction of antiviral genes and proinflammatory cytokines by HM-*γ*-PGA in RAW 264.7 cells. (a) Cells were treated with medium only, HM-*γ*-PGA (1 mg/mL), and 100 ng/mL of LPS. The time-dependent changes in mRNA expression after treatment were confirmed by PCR. All samples were normalized using GAPDH wherein equal amounts PCR products were run on 1.5% ethidium bromide impregnated agarose gels and visualized using GelDoc Imaging System. (b) RBI (Gene/GAPDH) of the IFN-*β*, IRF-3, IRF-7, and IFN-related genes of Figure (a). RBI was determined using GelDoc Imaging System Band Quantification Software. Error bars indicate the range of values obtained from two independent experiments.

**Figure 3:**
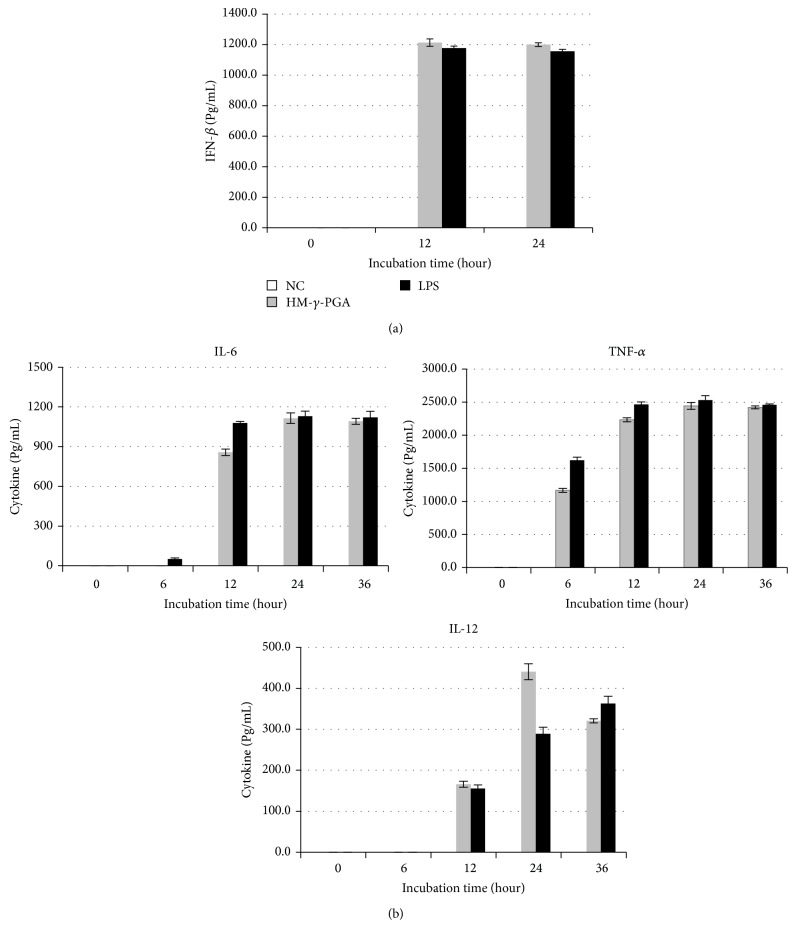
Ability of HM-*γ*-PGA to induce proinflammatory cytokine secretion in murine macrophage cell line. Cells were treated with medium only, HM-*γ*-PGA (1 mg/mL), and 100 ng/mL of LPS. The time-dependent changes in cytokine secretion after treatment were determined by ELISA (a) IFN-*β*; (b) proinflammatory cytokines. The data show representative means ± SD of each murine cytokine measured over time from three independent assays.

HM-*γ*-PGA treated RAW 264.7 cells showed increased mRNA expression of interferon regulatory transcription factors 3 (IRF-3) and 7 (IRF-7) and IFN-stimulated genes such as myxovirus resistance protein 1 (Mx1) and interferon-induced guanylate-binding protein 1 (GBP1) almost comparable with the level induced by LPS beginning 3 hours posttreatment (Figure 2). However, IFN-*β* as well as interferon stimulated gene-15 (ISG-15) mRNA expression levels was not as high as compared to LPS treated cells, though still evidently higher in contrast to the negative control. Since mRNA expression does not necessarily correlate with the secreted protein levels, proinflammatory cytokine secretion was also measured after HM-*γ*-PGA stimulation via murine cytokine ELISA kits (Figure 3). The present research showed that HM-*γ*-PGA can induce cytokine secretion in RAW 264.7 cells comparable with the LPS treated cells.

## 4. Discussion

The capacity of NDV to cause a highly contagious infection resulting in high mortality and reduced farm efficiency remains to threaten the global poultry industry. Currently available vaccines cannot provide adequate immunity in poultry even with the use of multiple vaccinations [9]; hence natural substances which have antiviral activity are of great importance. One of the natural substances widely studied for its various biological functions and applications is the HM-*γ*-PGA, a natural, edible, and biodegradable polymer derived from* Bacillus subtilis* subsp.* chungkookjang* [10–12]. HM-*γ*-PGA is secreted from *γ*-PGA synthetase ABC complex on the wall of* Bacillus subtilis* subsp.* chungkookjang* [11]. This naturally secreted HM-*γ*-PGA is a safe and edible polymer which contains negligible toxins which do not interfere with its beneficial effects/applications such as satisfactory adjuvant function, antitumor effect, and innate immunity inducible role [14, 16]. Among its biological functions, its capacity to initiate immune responses in mice via TLR4 signaling just like LPS makes HM-*γ*-PGA a potent immunomodulator with a big potential as a therapeutic agent [12]. Briefly, TLRs are considered to be a major component of the pattern recognition system which detects invading pathogens by recognizing pathogen associated molecular patterns (PAMPs). And one of the well-studied TLRs is TLR4. Mammalian (mouse) TLR4 signaling pathway starts by the transfer of LPS-binding protein (LBP) of the detected LPS to the cluster of differentiation 14 (CD14) on the surface of inflammatory cells. This reaction eventually leads to the transfer of LPS to TLR-4 via myeloid differentiation-2 (MD-2) [24]. Successful activation of TLR4 initiates intracellular activation of myeloid differentiation primary response protein-88 (MyD88-) dependent and MyD88-independent pathways. MyD88-dependent pathway utilizes MyD88 and Toll-interleukin 1 receptor (TIR) domain-containing adapter protein (TIRAP) to successfully export nuclear factor-K*β* (NF-K*β*) to the nucleus to initiate transcription of proinflammatory cytokine genes, while MyD88-independent pathway makes use of Toll/interleukin-1 receptor-domain-containing adapter-inducing interferon-*β* (TRIF) and TRIF-related adapter molecule (TRAM) to effectively activate transcription factor IRF-3 and subsequent production of IFN-*β* [25]. Though proinflammatory cytokines such as TNF-*α*, IL-6, and IL-12 are important for a successful inflammatory response, induction of Type I interferon is much more considered to be indispensible for antiviral resistance. Nonetheless, TLR4 is considered to be unique among the TLR family in the fact that LPS recognition results in activation of both MyD88-dependent and the TRAM/TRIF-dependent signaling pathways [26].

In this study, HM-*γ*-PGA has been tested through several experiments to evaluate its application to control Newcastle disease virus replication on murine macrophage cells (RAW 264.7). Since the immune-stimulating effect of HM-*γ*-PGA has been well established in mice and murine macrophage cells [11–13, 16] and previous studies have already elucidated the interaction between NDV and murine macrophages [21, 27], we decided to use murine macrophages to evaluate the antiviral effect of HM-*γ*-PGA against NDV.

In the pretreatment antiviral assay, HM-*γ*-PGA treatment was able to reduce viral replication on NDV-GFP infected RAW 264.7 cells as shown by lower NDV M gene and GFP expressions and higher cell survivability after infection (Figure 1). However, coincubation of the virus with HM-*γ*-PGA and postinfection treatment with HM-*γ*-PGA did not reduce the gfp expression of the virus (see Supplemental Figure 1 in the Supplementary Material available online at http://dx.doi.org/10.1155/2014/301386).

Based on these findings, HM-*γ*-PGA has been hypothesized to be involved with the antiviral state in cells via induction of Type I interferons. The previous study showed that the expression of antiviral effector molecules protein kinase R (PKR), 2′-5′-ologoadenylate synthetase (OAS), and Mx, as induced by Type 1 interferons, correlates with the susceptibility to NDV infection in both primary macrophages and macrophage-derived tumor cells (e.g., RAW cells) as these molecules inhibit critical steps during RNA translation and/or assembly of virus particles in viral replication [21]. In addition, ISG-15 protein, a ubiquitin-like modifier, greatly expressed after Type I interferon stimulation has been shown to mediate protection in a number of different viral infection models [28].

Likewise, the same paper also demonstrated that RAW cells have delayed IFN secretion after NDV infection thus making them susceptible to the said virus [21]. However, as compared to chicken cells, mammalian cells have innate resistance with the V protein of NDV which can block production of IFN [29]. On the other hand, Moon et al. showed that HM-*γ*-PGA can induce a significant amount of IFN-*β* in RAW 264.7 cells comparable with LPS induced IFN-*β* secretion as measured by murine cytokine ELISA [13]. With this in mind, the researchers hypothesized that the early induction of Type I interferon can also protect RAW 264.7 cells against NDV infection. In the present study, HM-*γ*-PGA treated Raw 264.7 cells showed increased mRNA expression of transcriptional factors IRF-3, IRF-7, and IFN-stimulated genes such as Mx1 and GBP1 almost comparable with the level induced by LPS beginning 3 hours posttreatment. However, IFN-B as well as ISG-15 mRNA expression levels was not as high as compared to LPS treated cells, though still evidently higher in contrast to the negative control. Such observations are in accordance with the findings of group of Wilden in 2009 that strong expression of antiviral genes IRF-3, IRF-7, and IFN-*β* and retinoic acid-inducible gene 1 (RIG-I) can provide protection against NDV infection [21]. Although RIG-1 was not measured in this study, the strong expression of IRF-3 and IRF-7 alone can strongly suggest that antiviral state has been achieved in HM-*γ*-PGA treated RAW 264.7 cells since these transcriptional factors are indispensable for the induction of Type I IFNs [30].

However, since mRNA expression does not necessarily correlate with the secreted protein levels, proinflammatory cytokine secretion was also measured. Using commercial murine cytokine ELISA kits for detection of IL-6, IL-12, TNF-*α*, and IFN-*β*, the present research showed that HM-*γ*-PGA can induce cytokine secretion in RAW 264.7 cells comparable with the LPS treated cells. Nevertheless, overstimulation of the innate immunity can possibly lead to excessive cytokine release which can be detrimental to the cells. This study is no exception with the possibility of a cytokine storm after treatment of an immune-stimulating agent, HM-*γ*-PGA, and infection of NDV, a virus known to aberrantly affect cytokine responses. Based on the limited results of the study, pretreatment of murine macrophage cells prior to NDV infection only resulted in less than 10% cell death in contrast to more than 50% of the medium treated cells. Assuming that excessive cytokine release indeed occurred in cells pretreated with HM-*γ*-PGA, the results still suggest that the upregulation of cytokines in this study did not adversely affect the treated cells as compared to medium treated cells. Thus we can observe that pretreatment of HM-*γ*-PGA at the appropriate time prior to NDV infection provided protection rather than death to the susceptible cells.

Moreover, since we did not test the protein expression levels of the interferon stimulated antiviral proteins Mx, OAS, PKR, and ISG 15, we could only speculate that these proteins have been sufficiently expressed and successfully inhibited virus replication; thus pathogen recognition receptors (PRRs) were not maximally stimulated, preventing a possible cytokine storm. Therefore, our findings suggest that HM-*γ*-PGA, if given at the appropriate time prior to NDV-GFP infection, can be a significant antiviral substance which can inhibit NDV infection through its stimulation of antiviral state on RAW 264.7 cells.

Though the experiments were done on cells not normally infected by NDV, the promising results of the present study serve as justification for conducting tests on the applicability of HM-*γ*-PGA in chicken immune cells. Nonetheless, our lab has already started the evaluation of the anti-NDV activity of HM-*γ*-PGA in both chicken and chicken immune cells, to fully assess the applicability of HM-*γ*-PGA in the poultry industry.

## Supplementary Material

“The virucidal and post-infection treatment assays of HM-γ-PGA against NDV-GFP.”

## Figures and Tables

**Table 1:** Primer sets used to quantify viral RNA expression.

Genes	Primers	
	Forward	Reverse
APMV-1 M gene	5′-AGTGATGTGCTCGGACCTTC-3′	5′-CCTGAGGAGAGGCATTTGCTA-3′
Murine GAPDH	5′-TGACCACAGTCCATGCCATC-3′	5′-GACGGACACATTGGGGGTAG-3′

**Table 2:** Primer sets used to quantify antiviral gene mRNA expression.

Genes	Primers	
	Forward	Reverse
GAPDH	5′-TGACCACAGTCCATGCCATC-3′	5′-GACGGACACATTGGGGGTAG-3′
IFN-β	5′-TCCAAGAAAGGACGAACATTCG-3′	5′-TGCGGACATCTCCCACGTCAA-3′
Mx1	5′-GATCCGACTTCACTTCCAGATGG-3′	5′-CATCTCAGTGGTAGTCAACCC-3′
IRF-3	5′-GTGCCTCTCCTGACACCAAT-3′	5′-CCAAGATCAGGCCATCAAAT-3′
IRF-7	5′-AAGCTGGAGCCATGGGTATG-3′	5′-GACCCAGGTCCATGAGGAAG-3′
ISG15	5′-CAATGGCCTGGGACCTAAA-3′	5′-CTTCTTCAGTTCTGACACCGTCAT-3′
GBP1	5′-AAAAACTTCGGGGACAGCTT-3′	5′-CTGAGTCACCTCATAAGCCAAA-3′
