# Supplementary figures and images for: The Antiviral Effect of High-Molecular Weight Poly-Gamma-Glutamate against Newcastle Disease Virus on Murine Macrophage Cells
Source: Adv Virol. 2014 Dec 30;2014:301386. doi: 10.1155/2014/301386 (PMC4293867; doi:10.1155/2014/301386)

# Supplemental Figure 1

(A)

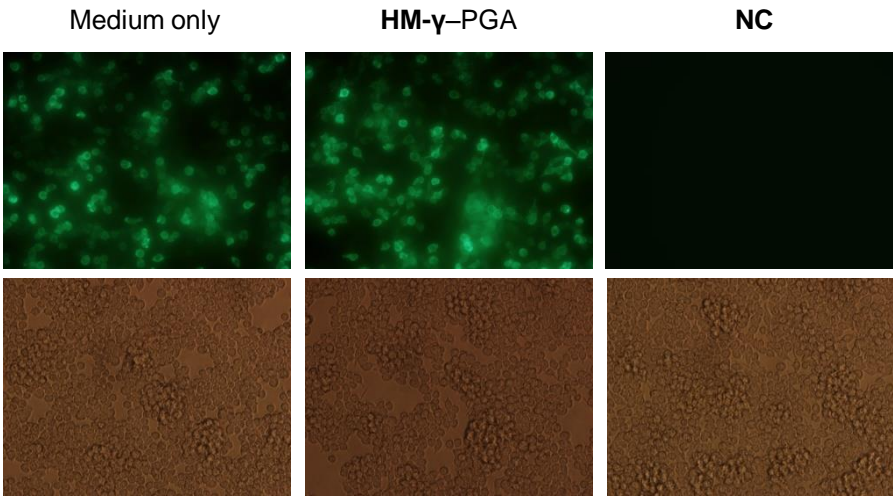

(B)

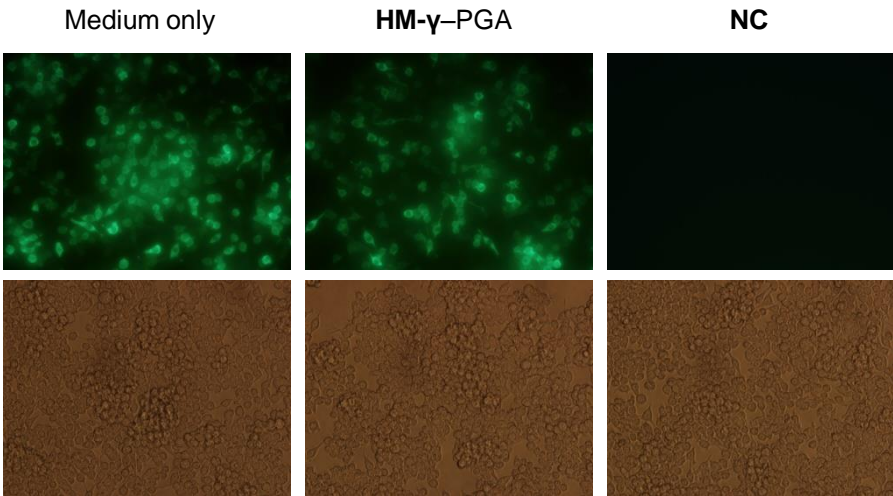

Supplement: Supplementary file 1 — “The virucidal and post-infection treatment assays of HM-γ-PGA against NDV-GFP.” [file 301386.f1.pdf]
